# Tai Chi as a preventive intervention for improving mental and physical health in non-depressed college students with high perceived stress

**DOI:** 10.3389/fpubh.2025.1613384

**Published:** 2025-11-12

**Authors:** Jingyu Sun, Ke Yao, Rongji Zhao, Hanfei Li, Antonio Cicchella

**Affiliations:** 1 Sports and Health Research Center, Department of Physical Education, Tongji University, Shanghai, China; 2 International College of Football, Tongji University, Shanghai, China; 3 Department for Quality-of-Life Studies, Bologna University, Bologna, Italy

**Keywords:** Tai Chi exercise, high perceived stress, non-depressed, mental and physical outcomes, college students

## Abstract

**Aim:**

Non-depressed college students with high perceived stress represent a distinct preclinical population at elevated risk for psychological deterioration. While Tai Chi is established as a mind–body exercise for improving well-being in clinical and older populations, its targeted efficacy in this specific at-risk subgroup—particularly regarding integrated physical and mental health benefits—remains inadequately explored. This study aimed to investigate the effects of a 16-week Tai Chi program on comprehensive fitness outcomes in this population.

**Methods:**

Eighty-eight non-depressed students with high perceived stress (Perceived Stress Scale scores between 38 and 56, Self-Rating Depression Scale scores below 50) were randomly assigned to a Tai Chi group (*n* = 47), which underwent a 16-week supervised program (3 sessions/week, 90 min/session), or a control group (*n* = 41) that maintained usual activities. Assessments pre- and post-intervention covered health-related physical fitness and mental health status (Perceived Stress Scale, Self-Rating Depression Scale, Pittsburgh Sleep Quality Index, Fatigue Scale-14, Hamilton Anxiety Scale, and SF-36).

**Results:**

Compared to controls, the Tai Chi group showed significant between-group improvements in lower-limb muscular endurance (squat test), perceived stress, sleep quality, somatic anxiety, role limitations due to physical health, and social functioning (all *p* < 0.05). Significant enhancements were also observed in physical functioning, fatigue, and general mental health (all *p* < 0.05).

**Conclusion:**

The 16-week Tai Chi intervention yielded concurrent benefits in physical and mental health among non-depressed college students with high perceived stress. These findings support Tai Chi as a feasible, multi-targeted preventive strategy against stress-related morbidity in this at-risk campus population.

**Clinical trial registration:**

Identifier ChiCTR2400089594, http://www.chictr.org.cn/index.html.

## 1 Introduction

College students navigate a critical developmental transition, facing a confluence of academic pressures, career uncertainties, and complex social dynamics that significantly elevate their risk for chronic stress (1, 2). The high prevalence of perceived stress and anxiety in this population is a global concern, with reported rates ranging from 32% to over 55% (3, 4), a trend exacerbated by recent societal challenges such as the COVID-19 pandemic (5, 6). Chronic exposure to such stressors is not merely a subjective discomfort; it is robustly linked to measurable declines in sleep quality, physical health, and psychological well-being, thereby increasing the susceptibility to clinical disorders like anxiety and depression (7). Consequently, the World Health Organization has identified stress management as a paramount public health priority for student populations (8).

Within this broad context, a specific preclinical subgroup warrants particular attention: non-depressed students experiencing high perceived stress. These individuals, while not meeting the clinical threshold for depression, represent an at-risk population in the prodromal stage of potential psychological deterioration (9, 10). Sustained high stress is a known correlate of depression pathogenesis, potentially inducing neuroplastic alterations that pave the way for depressive onset (11). For this subgroup, prolonged stress already impairs daily functioning and academic performance (12, 13), creating a vulnerable state that, without timely intervention, can escalate to major depressive disorder or other adverse outcomes (9, 14). This positions them as a critical target for early, preventive interventions aimed at curbing the progression along the stress-depression continuum.

Exercise is widely recognized as a viable non-pharmacological intervention to mitigate stress-related risks (15). Meta-analytic evidence supports the efficacy of aerobic exercise in reducing depressive symptoms in student populations (16). Among various forms, Tai Chi, a low-intensity mind–body aerobic exercise, demonstrates particular promise due to its unique integration of physical activity with meditative focus and diaphragmatic breathing (17). A growing body of evidence documents its benefits for multidimensional effects, including physical, psychological and quality of life benefits (17–22). Systematic reviews and randomized controlled trials, conducted mainly in older adults and clinical populations, indicate that Tai Chi consistently improves functional fitness (including BMI, body fat, vital capacity, lower limb strength, balance/dynamic stability, and flexibility) and yields cardiometabolic benefits, including reductions in blood pressure and improvements in glycemic control (23). In parallel, its mental health benefits, such as reducing stress (17), anxiety (18), and depressive symptoms (24), are attributed to its proposed mechanisms of regulating autonomic function (e.g., reducing cortisol, increasing heart rate variability) and fostering psychological self-regulation through mindful movement (17, 23–29).

However, a significant literature gap remains. While the effects of Tai Chi are well-documented in older or clinically ill cohorts, its mechanisms and efficacy are underexplored in the specific, preclinical subgroup of non-depressed college students with high perceived stress. This population faces a distinct set of challenges and possesses different resilience resources compared to older or clinical groups. The question of whether Tai Chi’s documented physical and mental benefits can be translated to confer preventive, dual-domain protection in this young, high-risk, yet non-clinical population has not been systematically investigated. Their underrepresentation in targeted exercise intervention research highlights a missed opportunity for early prevention.

To address this gap, the present study aimed to examine the effects of a 16-week Tai Chi intervention on a comprehensive set of mental and health-related physical fitness outcomes within this target population. We hypothesized that Tai Chi would yield significant improvements in both domains compared to a control group. Furthermore, the study concurrently evaluated its feasibility as a practical, campus-based stress management tool. By pursuing these objectives, this research provides novel insights into Tai Chi’s preventive potential against stress-related morbidity in higher education contexts, offering empirical support for institutional well-being initiatives.

## 2 Methods

### 2.1 Participants

All interventions were approved by the University Ethics Committee (2021tjdx024). Participants were recruited via flyers, social media, and printed notices posted on campus. A total of 116 respondents were initially invited to visit the research department. The study protocol and objectives were explained in detail to all potential participants prior to data collection. Written informed consent was obtained from all participants before registration.

**Figure 1:**
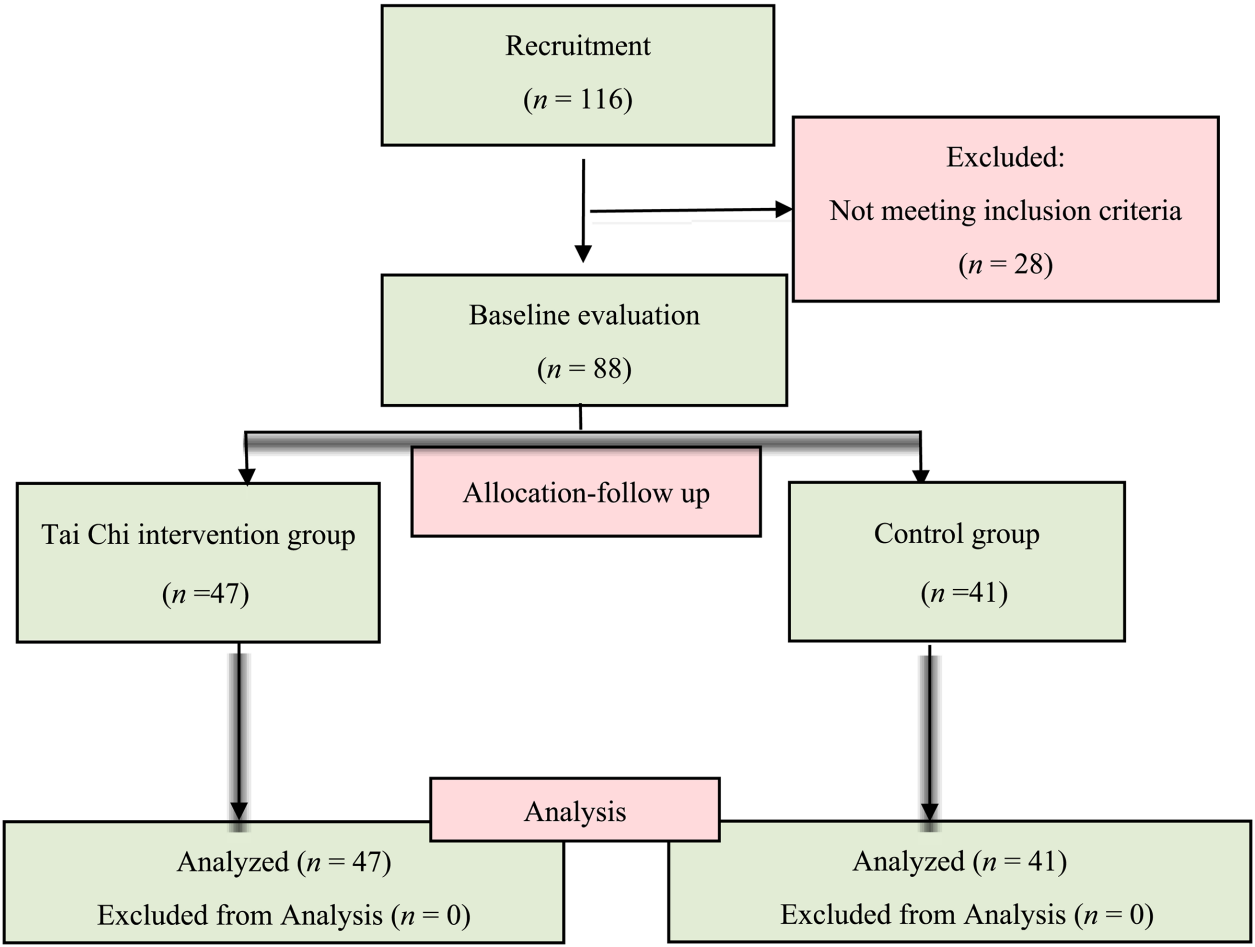
Participant flow diagram.

Participants were all undergraduate students from colleges, aged between 18 and 23. Participants were subjected to an initial screening that included completion of questionnaires to collect background information (e.g., sedentary behavior, medical history, current health status, etc.), and a physical examination. Based on the following inclusion exclusion criteria: (1) participants aged 18–23 years; (2) Perceived Stress Scale (PSS) scores between 38 and 56, confirming high perceived stress status (30); (3) Self-Rating Depression Scale (SDS) scores below 50 to ensure absence of depression; and (4) Kessler Psychological Distress Scale (K10) scores below 30 (excluding severe psychological distress), thereby maintaining relative psychological homogeneity in the sample and minimizing potential confounding effects of extreme psychological distress on primary outcomes; (5) Willing to accept the principle of randomization; (6) Willing to take the tests and sign an informed consent form. Those participants who satisfied the following criteria were excluded: (1) Suffering from a disease that affects their athletic performance; (2) Had a history of heart disease, severe arrhythmia, or pacemaker use; (3) Regular smokers or heavy drinkers of alcohol or other stimulating beverages; and (4) Taking medications that may affect their emotional responses, such as anxiolytics or antidepressants, at least 1 month before this study. A total of 88 students were eventually retained in the experiment (Figure 1).

### 2.2 Experimental procedures

Participants completed baseline assessments consisting of demographic questionnaires and standardized psychometric instruments: Pittsburgh Sleep Quality Index (PSQI), Hamilton Anxiety Scale (HAMA), the 14-item Fatigue Scale (FS-14) and 36-Item Short Form Health Survey (SF-36). Health-related physical fitness evaluations included eight standardized measures: (1) body weight and BMI, (2) resting heart rate, (3) vital capacity, (4) blood pressure, (5) one-minute sit-up test, (6) standing long jump, (7) one-minute squat test, and (8) eyes-closed single-leg balance test.

Sample size was determined from prior evidence (PMID: 25686304) indicating that a standardized mindful awareness practices intervention produced significant improvements in sleep quality, a stress-related outcome. Using G*Power for a two-tailed test (*α* = 0.05, Cohen’s d = 0.80), we estimated that 26 participants per group would be required. To ensure adequate power, we set a target sample size of 88 and subsequently randomized 88 university students to either a Tai Chi intervention group (*n* = 47), assigned to regular 24-form Yang-style Tai Chi practice, or a control group (*n* = 41) instructed to maintain their usual lifestyles (19). The intervention group received 4 weeks of foundational training from a senior Tai Chi instructor (20 years’ teaching experience), followed by a structured 16-week intervention (3 sessions/week, 90 min/session). Each session comprised: (1) 10-min warm-up (gentle stretching and breathing exercises), (2) 70-min core Tai Chi practice, and (3) 10-min cool-down (meditation and slow walking). All participants resided within the same closed-campus community. Adherence was ensured through dual oversight: a certified Tai Chi master/research assistant supervised all sessions, and participants signed standardized attendance sheets at every meeting.

### 2.3 Health-related physical fitness measurement

All assessments were administered between 14:00 and 17:00 in a temperature-controlled laboratory (24–26 °C) under standardized lighting. Participants abstained from caffeine and alcohol for at least 24 h before testing and rested quietly for at least 10 min upon arrival. To minimize fatigue carryover, tests followed a fixed sequence: (1) anthropometrics and vitals, (2) lung function, (3) balance, (4) muscular strength/power, and (5) muscular endurance. Unless otherwise specified, three trials were permitted with 1–2 min of passive rest between trials and ~3 min between different items; the best performance was retained for analysis.

Pulmonary function was indexed by forced vital capacity (FVC) (31), measured with a portable spirometry device (HK6000/6800 FH; Hengkang Jiaye, Shenzhen, China). During testing, participants grasped the mid-section of the mouthpiece with both hands, refrained from placing their lips on the tube during inspiration, and took care not to occlude the air outlet with their hands while exhaling.

Muscle strength was assessed using handgrip dynamometry and the standing long jump (SLJ) (32). Maximal voluntary grip strength was obtained with an electronic dynamometer (CAMRY-EH101; Hengkang Jiaye, Shenzhen, China) across three trials per hand. The SLJ was performed three times, and the greatest distance achieved was retained for analysis.

Muscular endurance was evaluated with one-minute sit-up and one-minute squat tests (33). For sit-ups, participants lay supine on a mat with feet at shoulder width, knees flexed, and arms crossed over the chest; a valid repetition required elbow-to-knee contact on the ascent and the shoulder blades touching the mat on the descent. The count of correct repetitions completed within 60 s was documented. For squats, participants adopted a natural stance with feet externally rotated about 30°. Throughout the movement, knees did not travel beyond the toes and were aligned with the direction of the toes; repetitions completed in 1 min were tallied.

Static balance was examined using a single-leg stance with eyes closed (34). Barefoot on a level surface, participants elevated one leg—maintaining approximately a 90° bend—while the contralateral foot remained on the floor. They were instructed to keep an upright posture and minimize sway. The trial ended if the eyes opened, the lifted foot touched down, or the arms were used for support. Time to loss of balance was recorded, with longer durations indicating superior static balance.

### 2.4 Questionnaire assessments

All questionnaires were administered in their validated Chinese versions between 14:00 and 17:00 in a quiet laboratory under standardized lighting with trained proctor supervision. Participants rested quietly for at least 10 min before testing and received uniform instructions. To reduce respondent burden and maintain consistency, a fixed sequence was used: (1) PSS, (2) SDS, (3) PSQI, (4) HAMA, (5) FS-14, and (6) SF-36. Brief intervals of approximately 1–2 min were provided between instruments. Proctors checked forms on site for completeness and clarity; any missing or multiple responses were corrected immediately before scoring.

This study employed internationally recognized standardized instruments—the PSS (35), SDS (36), PSQI (37), HAMA (38), FS-14 (39), and SF-36 (40)—all of which have well-documented reliability, validity, and responsiveness. These measures have been repeatedly used as primary or key secondary endpoints in randomized controlled trials and large cohort studies published in high-impact journals (41–45), thereby ensuring the scientific rigor, methodological standardization, and cross-study comparability of our outcome assessments. The PSS is a well-established psychometric instrument designed to evaluate an individual’s subjective experience of stress (35). Scores between 38 and 56 are classified as indicative of high stress, with progressively higher scores reflecting more severe perceived stress levels (46). The SDS quantifies the presence and intensity of depressive symptomatology (36). Each item is rated on a 4-point scale (1 = none or a little of the time to 4 = most or all of the time), yielding a total score from 20 to 80, with higher scores indicating greater symptom severity (36). PSQI score is derived from the sum of seven component scores, yielding a total ranging from 0 to 21 (47). Lower scores reflect better sleep quality, while higher scores indicate poorer sleep. A score above 5 typically signifies significant sleep disturbances (48). The HAMA widely applied in clinical and research settings, assesses anxiety severity across both psychological and somatic domains (49). The instrument comprises 14 items graded from 0 to 4, where 0 denotes no anxiety and 4 indicates marked severity. The aggregate score ranges from 0 to 56, providing a quantitative index of anxiety burden. Conventional interpretive bands are: values >17 suggest mild anxiety; scores of 17–23 indicate moderate anxiety; and scores of 25–30 denote severe anxiety (50). The FS14 is a fatigue perception questionnaire that assesses two key dimensions: physical fatigue and psychological fatigue. The total score ranges from 0 to 33, with higher scores indicating greater fatigue severity (51). The SF-36 is a widely used instrument for assessing health-related quality of life, measuring self-reported physical and mental health across eight domains (52): physical functioning (PF), role limitations due to physical health (RP), bodily pain (BP), general health perceptions (GH), vitality (VT), social functioning (SF), role limitations due to emotional problems (RE), and mental health (MH). Additionally, this questionnaire was utilized to evaluate changes in perceived health status over the preceding year (53).

Questionnaires were administered in their validated Chinese versions (50, 51, 54–58).

### 2.5 Statistical analysis

Statistical analyses of all data were performed using the SPSS statistical package (IBM SPSS Statistics, version 25). Extracted data included health-related physical fitness measures (anthropometrics and vitals, lung function, balance, muscular strength/power, and muscular endurance) and questionnaire scores (PSS, SDS, PSQI, HAMA, FS-14, and SF-36). For each outcome, means and standard deviations (SD) were calculated at baseline and after the 16-week intervention. Levene’s and Shapiro–Wilk tests were used to check the homogeneity of variance and normality of data, respectively. Between-group differences in change scores (Δ = Baseline – Post) were examined with independent-samples *t*-tests, and within-group pre–post changes were evaluated with paired *t*-tests for both physical and psychological outcomes. Two-sided *p*-values with 95% confidence intervals are reported, and statistical significance was set at *α* = 0.05.

## 3 Results

### 3.1 Baseline characteristics of non-depressed college students with high perceived stress

There was no significant difference in age (*p* = 0.062), sex distribution (*p* = 0.45), PSS (*p* = 0.31), SDS (*p* = 0.16) and K10 (*p* = 0.72) between the two groups (Table 1).

**Table 1:** Summary of basic characteristics of high perceived stress in non-depressed college students with high perceived stress.

Index		Control group	Intervention group	*p*
Gender	Male	22 (53.7%)	29 (61.7%)	0.45
	Female	19 (46.3%)	18 (38.3%)	
Age		19.34 ± 1.04	18.28 ± 0.97	0.062
Weight Status	Lean	*n* = 6	*n* = 2	
	Normal	*n* = 17	*n* = 26	
	Overweight	*n* = 13	*n* = 14	
	Obese	*n* = 5	*n* = 5	
		Total: 41	Total: 47	
PSS		44.95 ± 3.63	44.85 ± 2.31	0.31
SDS		37.59 ± 9.11	41.17 ± 5.04	0.16
K10		20.12 ± 6.63	19.66 ± 5.47	0.72

Lean: BMI <18.5, Normal: BMI 18.5–23.9, Overweight: BMI 24–28, Obese: BMI>28.

### 3.2 Effects of tai chi exercise on physical outcomes in non-depressed college students with high perceived stress

As shown in Table 2, within-group comparisons showed significant changes in the Tai Chi intervention group for the 1-min deep-squat test (*p* < 0.05); However, the participants in the control group did not show such changes (*p* > 0.05).

**Table 2:** Changes in mental indicators of the participants in the control and intervention groups.

Index		Control (*n* = 41)			Intervention (*n* = 47)			*p*
		Pre	Post	Change (95% CI)	Pre	Post	Change (95% CI)	
General physiological indicator	Weight (kg)	69.17 ± 13.86	68.57 ± 14.58	0.59 (−0.32 to 1.51)	64.81 ± 12.67	64.25 ± 13.05	0.56 (−0.05 to 1.18)	0.96
	BMI (kg/m^2^)	23.00 ± 3.67	23.10 ± 3.65	−0.10 (−0.25 to 0.06)	23.50 ± 3.35	23.44 ± 3.36	0.06 (−0.07 to 0.19)	0.12
	HR (points)	84.00 ± 13.85	80.66 ± 13.81	3.34 (−1.34 to 8.02)	84.43 ± 14.44	80.62 ± 9.99	3.81 (−0.37 to 7.99)	0.88
	Vital Capacity (mL)	3402.24 ± 747.80	3404.83 ± 750.13	−2.59 (−114.54 to 109.37)	3438.83 ± 789.83	3457.43 ± 797.20	−18.60 (−110.20 to 73.01)	0.82
Blood pressure	SBP (mmHg)	122.88 ± 18.87	123.05 ± 19.02	−0.17 (−0.75 to 0.41)	116.55 ± 13.34	117.13 ± 13.56	−0.58 (−1.17, 0.02)	0.33
	DBP (mmHg)	75.27 ± 8.58	75.21 ± 8.64	0.05 (−0.27 to 0.36)	74.77 ± 8.20	74.68 ± 8.08	0.09 (−0.14 to 0.31)	0.85
Upper limb muscle strength	RHGS (kg)	31.94 ± 10.38	33.20 ± 9.47	−1.26 (−2.29 to −0.23)	31.95 ± 9.14	31.90 ± 9.24	0.05 (−1.19 to 1.29)	0.11
	LHGS (kg)	29.26 ± 9.99	30.00 ± 10.24	−0.73 (−1.79 to 0.32)	30.08 ± 8.98	29.61 ± 8.86	0.47 (−0.38 to 1.32)	0.07
Lower limb muscle strength	SLJ (points)	188.27 ± 33.46	186.66 ± 30.79	1.61 (−1.15 to 4.37)	193.21 ± 42.42	191.74 ± 39.95	1.47 (−1.38 to 4.31)	0.94
	1-min Squat (number)	43.51 ± 6.94	43.54 ± 8.13	−0.02 (−2.06 to 2.00)	47.34 ± 8.31	50.09 ± 8.23 ^**^	−2.74 (−4.18 to −1.31) ^#^	0.03
Muscular endurance	1-min sit-up (number)	34.39 ± 8.13	34.22 ± 8.37	0.17 (−0.16 to 0.50)	33.34 ± 9.04	33.36 ± 9.15	−0.02 (−0.31 to 0.26)	0.37
Static balance	Balance (second)	39.32 ± 24.66	42.02 ± 25.09	−2.71 (−5.84 to 0.43)	45.72 ± 31.77	45.34 ± 29.65	0.38 (−2.18 to 2.94)	0.12

Data are presented as Mean ± SD. ^*^p < 0.05 and ^**^p < 0.01, intragroup comparison. ^#^*p* < 0.05 and ^##^*p* < 0.01, between-groups comparison at the same time. p value indicates differences in changes between the control and intervention group. Weight, Body Weight; BMI, Body Mass Index; SBP, Systolic Blood Pressure; DBP, Diastolic Blood Pressure; HR, Heart Rate; LHGS, Left-hand Grip Strength; RHGS, Right-hand Grip Strength; 1-min Sit-Up, 1-Minute Sit-Up Test; SLJ, Standing Long Jump; 1-min Squat, 1-Minute Squat Test.

The between-group comparisons indicated that, following the 16-week Tai Chi intervention, participants in the intervention group exhibited a significant improvement in lower-limb muscle strength, as evidenced by a significant increase in the number of deep-squat repetitions completed in 1 min (*p* < 0.05).

### 3.3 Effects of tai chi exercise on mental outcomes in non-depressed college students with high perceived stress

As shown in Table 3, within-group comparisons revealed significant improvements in the Tai Chi intervention group in perceived stress scores measured by the PSS questionnaire, sleep quality scores assessed by the PSQI questionnaire, body anxiety scores from the HAMA questionnaire, physical and mental fatigue scores as well as total scores in the FS-14 questionnaire, and physical functioning, social functioning, role limitations due to physical health, and mental health scores in the SF-36 questionnaire (*p* < 0.05). In contrast, no significant changes were observed in the control group (*p* > 0.05).

**Table 3:** Changes in mental indicators of the participants in the control and intervention groups.

Index		Control (*n* = 41)			Intervention (*n* = 47)			*p*
		Pre	Post	Change (95% CI)	Pre	Post	Change (95% CI)	
Perceived stress		44.73 ± 3.67	46.09 ± 3.23	−1.36 (−3.15 to 0.43)	45.43 ± 2.72	43.23 ± 2.83 ^**^	2.19 (1.03 to 3.35) ^##^	0.00
Depression		40.37 ± 4.65	41.62 ± 11.22	−1.26 (−4.61 to 2.10)	41.83 ± 5.01	42.02 ± 7.07	−0.19 (−2.38 to 2.00)	0.58
Sleep quality (PSQI score)		5.02 ± 2.62	5.41 ± 2.90	−0.39 (−1.29 to 0.51)	5.72 ± 2.64	3.96 ± 1.60 ^**^	1.77 (0.99 to 2.54) ^##^	0.00
Anxiety (HAMAscore)	HAMA/Mental (points)	6.80 ± 4.95	5.76 ± 3.56	1.04 (−0.42 to 2.51)	6.32 ± 4.90	6.40 ± 3.86	−0.09 (−1.31 to 1.14)	0.23
	HAMA/Somatic (points)	4.51 ± 3.03	4.34 ± 3.37	0.17 (−0.94 to 1.28)	4.36 ± 3.42	2.89 ± 2.57^**^	1.47 (0.77 to 2.17) ^#^	0.04
	HAMA/Total (points)	11.32 ± 6.15	10.10 ± 5.47	1.22 (−0.76 to 3.19)	10.68 ± 6.00	9.30 ± 4.94	1.38 (0.00 to 2.76)	0.89
Fatigue (FS14 score)	FS14/Physical (points)	4.76 ± 1.79	4.66 ± 1.49	0.10 (−0.49 to 0.69)	5.04 ± 2.24	4.23 ± 2.21^**^	0.81 (0.29 to 1.33)	0.07
	FS14/Mental (points)	2.59 ± 1.90	2.32 ± 1.92	0.27 (−0.26 to 0.80)	2.15 ± 1.71	1.66 ± 1.52^*^	0.49 (0.05 to 0.93)	0.52
	FS14/Total (points)	7.34 ± 2.41	6.98 ± 2.43	0.37 (−0.31 to 1.04)	7.19 ± 3.00	5.89 ± 2.70^**^	1.30 (0.56 to 2.04)	0.06
Health (SF36 score)	SF36/PF (points)	91.34 ± 10.96	93.54 ± 7.92	−2.20 (−5.15 to 0.76)	90.96 ± 9.48	95.21 ± 7.14 ^**^	−4.26 (−6.80 to −1.71)	0.29
	SF36/RP (points)	72.56 ± 30.00	68.90 ± 30.51	3.66 (−6.71 to 14.03)	76.06 ± 29.00	88.30 ± 17.17 ^**^	−12.23 (−20.31 to −4.15) ^#^	0.02
	SF36/BP (points)	78.80 ± 13.37	79.44 ± 12.91	−0.63 (−4.78 to 3.51)	82.40 ± 8.46	81.04 ± 11.33	1.36 (−1.92 to 4.64)	0.44
	SF36/GH (points)	55.34 ± 12.07	53.51 ± 8.63	1.83 (−1.92 to 5.58)	53.55 ± 9.07	55.64 ± 9.43	−2.09 (−5.15 to 0.98)	0.10
	SF36/VT (points)	60.73 ± 16.98	64.02 ± 18.34	−3.29 (−8.95 to 2.36)	62.13 ± 19.50	61.19 ± 19.05	0.94 (−4.30 to 6.17)	0.27
	SF36/SF (points)	86.99 ± 15.50	86.72 ± 13.88	0.27 (−4.78 to 5.32)	85.82 ± 16.00	92.43 ± 11.37 ^**^	−6.62 (−11.22 to −2.02) ^#^	0.045
	SF36/RE (points)	46.34 ± 37.92	59.35 ± 41.84	−13.01 (−28.05 to 2.03)	51.06 ± 41.03	51.06 ± 44.42	0.00 (−14.86 ± 14.86)	0.22
	SF36/MH (points)	71.02 ± 13.08	74.63 ± 15.58	−3.61 (−9.02 to 1.80)	71.64 ± 16.72	78.98 ± 15.55 ^*^	−7.34 (−13.25 to −1.43)	0.35

Data are presented as Mean ± SD. ^*^p < 0.05 and ^**^p < 0.01, intragroup comparison. ^#^*p* < 0.05 and ^##^*p* < 0.01, between-groups comparison at the same time. *p* value indicates differences in changes between the control and intervention group. Fatigue Scale-14; FS14/Physical, Physical Fatigue of Fatigue Scale-14; FS14/Mental, Mental Fatigue of Fatigue Scale-14; 36-Item Short Form Health Survey; SF36/MH, Mental Health of MOS 36-Item Short Form Health Survey; SF36/PF, Physical Functioning of MOS 36-Item Short Form Health Survey; SF36/RP, Role-Physical of MOS 36-Item Short Form Health Survey; SF36/BP, Bodily Pain of MOS 36-Item Short Form Health Survey; SF36/GH, General Health of MOS 36-Item Short Form Health Survey; SF36/VT, Vitality of MOS 36-Item Short Form Health Survey; SF36/SF, Social Functioning of MOS 36-Item Short Form Health Survey; SF36/RE, Role-Emotional of MOS.

Between-group comparisons indicated that, following the 16-week Tai Chi intervention, participants in the intervention group demonstrated significant improvements in perceived stress, sleep quality, somatic anxiety, role limitations due to physical health, and social functioning, as reflected in the scores of the PSS, PSQI, the somatic anxiety subscale of the HAMA, and the role-physical and social functioning domains of the SF-36 (*p* < 0.05).

## 4 Discussion

**Figure 2:**
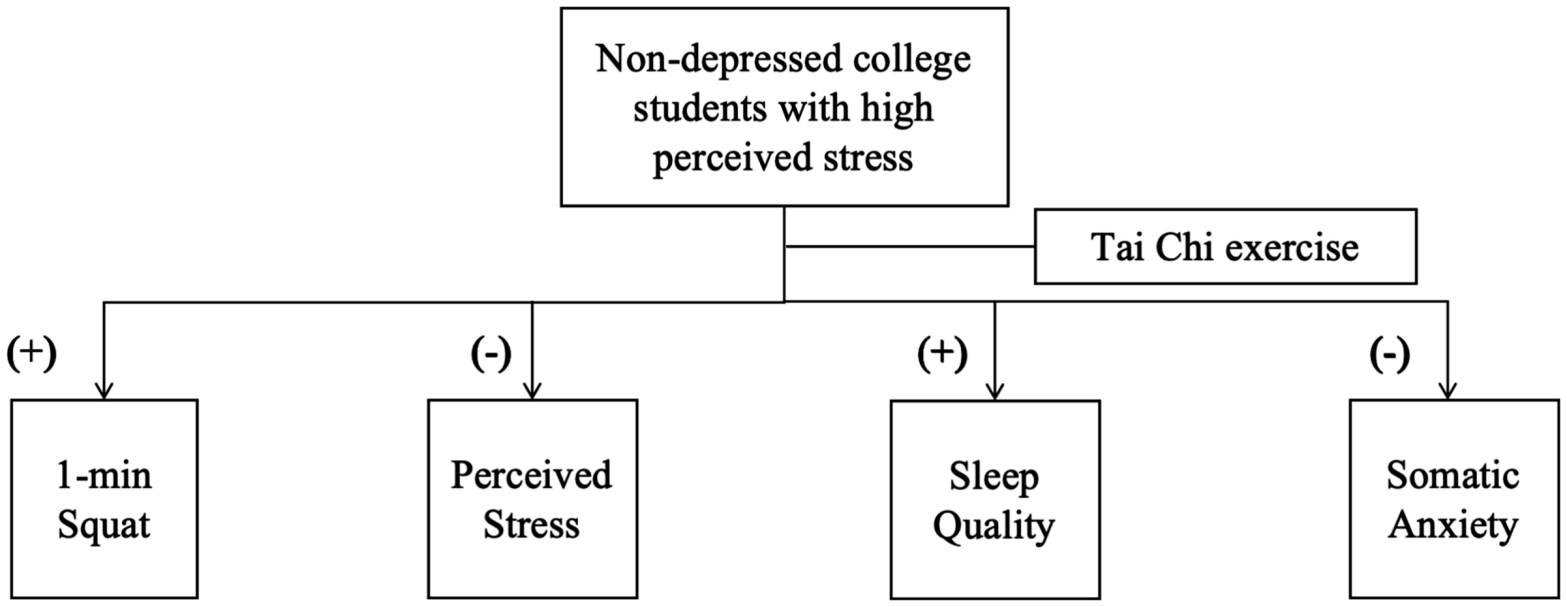
Tai Chi intervention effects in high-stress, non-depressed students.

This study demonstrates that a 16-week Tai Chi intervention led to significant and concurrent improvements across multiple health domains in non-depressed college students with high perceived stress—a preclinical subgroup at elevated risk for stress-related morbidity. As illustrated in Figure 2, participants in the Tai Chi group showed enhanced lower-limb muscular endurance (reflected by 1-min squat performance), reduced perceived stress (PSS), improved sleep quality (indicated by lower PSQI total scores), and decreased somatic anxiety (HAMA-Somatic subscale). These findings provide evidence supporting the utility of Tai Chi as an integrated intervention that concurrently enhances health-related physical fitness and alleviates key stress-related symptoms in this younger, non-clinical yet high-risk population, which has been underrepresented in previous research focusing primarily on older or clinical cohorts.

To interpret these findings, a multidimensional framework encompassing biomechanical, neurophysiological, and psychosocial pathways can be proposed. While the present design does not allow for causal inference, the interrelationships between these pathways offer a plausible, theoretical model for understanding Tai Chi’s potential effects. First, on a biomechanical level, the significant improvement in lower-limb muscular endurance is consistent with the demands of Tai Chi’s characteristic sustained, knee-flexed postures, which engage the quadriceps and related muscle groups (59). We speculate that this physical adaptation may be particularly beneficial for college students, as it could help counteract the physical fatigue and musculoskeletal discomfort that often accompanies chronic academic stress (60, 61). It is plausible that alleviating such somatic strain may, in turn, contribute to the observed reductions in global perceived stress and somatic anxiety, potentially by modulating the physical manifestations of stress.

Second, the observed reductions in perceived stress and somatic anxiety may be understood through neurophysiological perspectives. The integration of mindful movement, breath regulation, and meditative focus in Tai Chi is theorized to promote autonomic nervous system regulation, potentially favoring parasympathetic dominance (17, 62). Such a shift could be associated with the stabilization of physiological stress responses, offering a possible explanation for the parallel improvements in sleep quality (63). The fact that significant improvement was specific to somatic anxiety, rather than emotional anxiety (HAMA), might suggest that the intervention’s initial effects are more pronounced on the physiological and somatic level, possibly through enhancing body awareness and regulation, before significantly influencing core affective dimensions (64, 65).

Third, the enhancements in social functioning, and fewer physical health-related role limitations point to potentially important psychosocial processes. The group-based delivery of the intervention inherently created a context for social support and interaction, which may have been particularly beneficial for high-stress individuals who might otherwise experience social withdrawal (66). Furthermore, the process of mastering movement sequences and perceiving physical improvements may foster a strengthened sense of self-efficacy and perceived control—a process that could be termed “mind–body fortification” (66, 67). This enhanced self-efficacy might empower students to engage more fully in daily activities, thereby improving physical health-related role limitations and social functioning. The reduction in both physical and mental fatigue (FS-14) could be understood as a downstream effect of this process, where increased bodily stamina and the practice-induced relaxation may collectively alleviate exhaustion and its cognitive burden (68, 69).

Collectively, these findings position Tai Chi as a holistic intervention with relevance for the embodied experience of stress in a high-risk, preclinical student population. The co-occurrence of benefits across diverse domains suggests potentially synergistic effects. For instance, gains in physical endurance may support reductions in fatigue and somatic anxiety, which could then facilitate lower stress perception and greater social participation, thereby collectively building resilience. This profile highlights the innovative preventive value of Tai Chi as a campus-based strategy, specifically aimed at mitigating risk factors and promoting protective factors in a group that remains underrepresented in exercise intervention research, thereby potentially altering the trajectory toward more severe stress-related morbidity (62, 70, 71). Future research should prioritize longer-term trials to assess the sustainability of benefits and their impact on affective anxiety. Crucially, mechanistic studies incorporating physiological biomarkers and psychological mediators are needed to empirically validate the theoretical pathways proposed in this framework.

## 5 Limitation

Although this study provides valuable insights into the effects of Tai Chi training among non-depressed college students with high perceived stress, several limitations warrant acknowledgment. First, we recognize the multidimensional nature of stress assessment—while the PSS remains the gold standard for measuring perceived stress, future research would benefit from complementing PSS scores with physiological biomarkers (e.g., cortisol assays) or autonomic nervous system markers (e.g., heart rate variability) to achieve more comprehensive stress characterization, as noted in our revised discussion. Second, the single-intervention timepoint design limits our understanding of temporal effects; subsequent studies should incorporate multiple intervention timepoints to examine dose–response relationships. Future dose- and frequency-matched trials with active exercise comparators (e.g., brisk walking, flexibility training, or non-mindful calisthenics) are warranted to further distinguish Tai Chi–specific effects from general effects of physical activity. Third, the homogeneous sample of Chinese college students constrains generalizability, highlighting the need for cross-cultural validation across diverse ethnicities, regions, and populations (including non-depressed individuals with high stress from varying socioeconomic backgrounds). Finally, the underlying mechanisms through which Tai Chi improves psychological outcomes in this population remain unclear, necessitating multidisciplinary investigations integrating neurobiological, physiological, and psychological measures to elucidate the pathways of action.

## 6 Conclusion

This study investigated the effects of Tai Chi exercise on non-depressed college students experiencing high perceived stress—a preclinical population at elevated risk for psychological deterioration. The 16-week Tai Chi intervention resulted in significant improvements compared to the control group across multiple domains, including lower-limb muscular endurance (as assessed by the deep-squat test), perceived stress levels, sleep quality, somatic anxiety, and the role limitations due to physical health and social functioning domains of the SF-36 questionnaire. These findings provide robust evidence that Tai Chi is an effective, feasible campus-based intervention for enhancing both mental and health-related physical fitness in this high-risk student subgroup. By mitigating key stress-related impairments and fostering resilience, Tai Chi holds significant promise as a valuable public health strategy for preventing the progression toward stress-related morbidity, including depression, within college populations.

## Data Availability

The raw data supporting the conclusions of this article will be made available by the authors, without undue reservation.
